# Identifying chemopreventive agents for obesity-associated cancers using an efficient, 3D high-throughput transformation assay

**DOI:** 10.1038/s41598-019-46531-y

**Published:** 2019-07-16

**Authors:** Vanessa Benham, Blair Bullard, Thomas S. Dexheimer, Matthew P. Bernard, Richard R. Neubig, Karen T. Liby, Jamie J. Bernard

**Affiliations:** 1 Department of Pharmacology and Toxicology, Michigan State University, East Lansing, MI 48824 USA; 2 Division of Dermatology, Department of Medicine, College of Human Medicine, Michigan State University, East Lansing, MI 48824 USA

**Keywords:** Cancer prevention, Risk factors

## Abstract

Obesity is associated with ~40% of cancer diagnoses but there are currently no effective preventive strategies, illustrating a need for chemoprevention. We previously demonstrated that fibroblast growth factor 2 (FGF2) from adipose tissue stimulates malignant transformation, as measured by growth in soft agar, the gold-standard *in vitro* transformation assay. Because the soft agar assay is unsuitable for high throughput screens (HTS), we developed a novel method using 3D growth in ultra-low attachment conditions as an alternative to growth in agar to discover compounds that inhibit transformation. Treating non-tumorigenic, skin epithelial JB6 P^+^ cells with FGF2 stimulates growth in ultra-low attachment conditions analogous to growth in the soft agar. This transformation HTS identified picropodophyllin, an insulin growth factor 1 receptor (IGF1R) inhibitor, and fluvastatin, an HMG-CoA reductase inhibitor, as potential chemopreventive agents. These compounds were validated for efficacy using two non-tumorigenic cell lines in soft agar. Another IGF1R inhibitor and other statins were also tested and several were able to inhibit growth in soft agar. This novel 3D HTS platform is fast, robust and has the potential to identify agents for obesity-associated cancer prevention.

## Introduction

Obese individuals are a specific high-risk population that would benefit from targeted chemoprevention strategies. Obesity is associated with 13 different types of cancers and is associated with over 40% of cancer diagnoses^1^. Currently, 2 out of 3 American adults are considered either overweight or obese^2–4^. Rising obesity rates illustrate an immediate need for effective primary prevention strategies for obesity-associated cancers. Current prevention strategies like lifestyle changes are ineffective due to non-compliance. Although there are theories to explain the obesity-cancer association, the underlying mechanisms are poorly elucidated. While investigating this mechanism, our studies demonstrated that circulating fibroblast growth factor 2 (FGF2) from visceral adipose tissue (VAT) stimulates fibroblast growth factor receptor 1 (FGFR1) on epithelial cells to drive malignant transformation^5–7^. We demonstrated that VAT depleted of FGF2 failed to transform epithelial cells and epithelial cells lacking FGFR1, the primary receptor for FGF2, also failed to exhibit VAT-induced transformation^5^. Although FGF2 is considered a paracrine growth factor, its circulating levels correlate with adipose tissue mass in humans and are at levels sufficient to induce transformation *in vitro*^5,8^. This suggests that VAT-secreted FGF2 has a systemic role. In animals, we found that a high-fat diet increased serum FGF2 levels and that removing VAT via lipectomy depleted serum FGF2 levels^5^. Therefore, the FGF2/FGFR1 signaling axis is a potential chemopreventive target for obesity-associated epithelial cancers.

Compounds that lower cancer risk by delaying or preventing cancer, whether synthetic or natural, have the potential to significantly reduce cancer burden^9^. As the World Health Organization estimates that 30–50% of all cancer cases are preventable, lowering cancer incidence will in turn lower cancer mortality^10^. Effective chemopreventive agents either eliminate premalignant cells or protect normal/initiated cells from undergoing malignant transformation (i.e. the changes a non-transformed or normal cell undergoes to become carcinogenic)^10,11^. There are, however, drawbacks to implementing chemoprevention^12^. As these agents would benefit only a subset of the treated population, they must be efficacious and have minimal to no side effects so that they are tolerated for a long duration^13^. Cumulatively, these improbable standards are a challenge for the development and implementation of chemoprevention. Applying an approach of precision medicine to chemoprevention can help overcome these shortfalls^11^. Target-driven strategies to risk-stratify individuals would reduce strict restraints on side effects because the clinical benefits would presumably outweigh the risks^11^. Therefore, implementing targeted chemoprevention strategies in conditions such as obesity has the potential to greatly reduce cancer burden.

Methodological limitations make identifying compounds that prevent obesity-associated cancers challenging. Transformation is commonly modeled by the soft agar assay, a well-established technique that measures transformation by assessing anchorage-independent growth^14–18^. Non-transformed cells must be anchored to an extracellular matrix (ECM) or a treated-culture plate to proliferate. In contrast, tumorigenic cells, which have undergone transformation, lose their anchorage-dependence^19^. 3D models like the soft agar assay do not provide an ECM-like environment, so cells suspended in soft agar only proliferate and form colonies if they are transformed^19^. This gives 3D models a distinct advantage for modeling transformation over 2D culture methods and can distinguish transformed and non-transformed cells^19,20^. We previously used the soft agar assay to show that factors derived from VAT as well as FGF2 itself can stimulate epithelial cells to transform^5–7^. This experiment can identify chemopreventive compounds that prevent or inhibit FGF2-stimulated transformation, but the soft agar assay is unsuitable for high-throughput screening because it is laborious and inefficient, using a 6–24 well format with a 2-week incubation period.

Identifying compounds for precision chemoprevention requires a targeted high-throughput screening platform that models malignant transformation of non-transformed cells. However, this is an underdeveloped area of cancer prevention as current models use cancer cells lines and 2D culture. Currently, there are not any high throughput assays that model the process of transformation^19,20^. Therefore, the objective of these studies was to develop a high-throughput 3D model of transformation to screen for chemopreventive agents and then validate hits in soft agar with two cell lines, JB6 P^+^ and MCF-10A. These are, respectively, non-tumorigenic mouse epidermal cells and non-tumorigenic human breast epithelial cells. In a recent publication, Rotem *et al*. describe an HTS assay (384-well) where growth in ultra-low attachment conditions in a round bottom plate (3D growth) is strongly correlated to growth in soft agar^17^. Investigators measured growth of non-transformed and transformed cell lines in soft agar and in low attachment conditions and demonstrated a strong correlation with an R^2^ value of 0.82^17^. In this manuscript, we used growth in ultra-low attachment conditions to develop a novel phenotypic transformation HTS assay using FGF2/FGFR1 signaling as the target-based mechanism to identify chemopreventive agents for obesity-associated epithelial cancers. Screened compounds that prevent growth in ultra-low attachment conditions may further the mechanistic understanding of malignant transformation and have the potential to be developed as precision chemopreventive therapies. While this screen could identify novel inhibitors of FGF2/FGFR1 signaling, it also has the potential to find compounds that interrupt the transformation process itself.

## Results

### Development and optimization of transformation HTS

**Figure 1:**
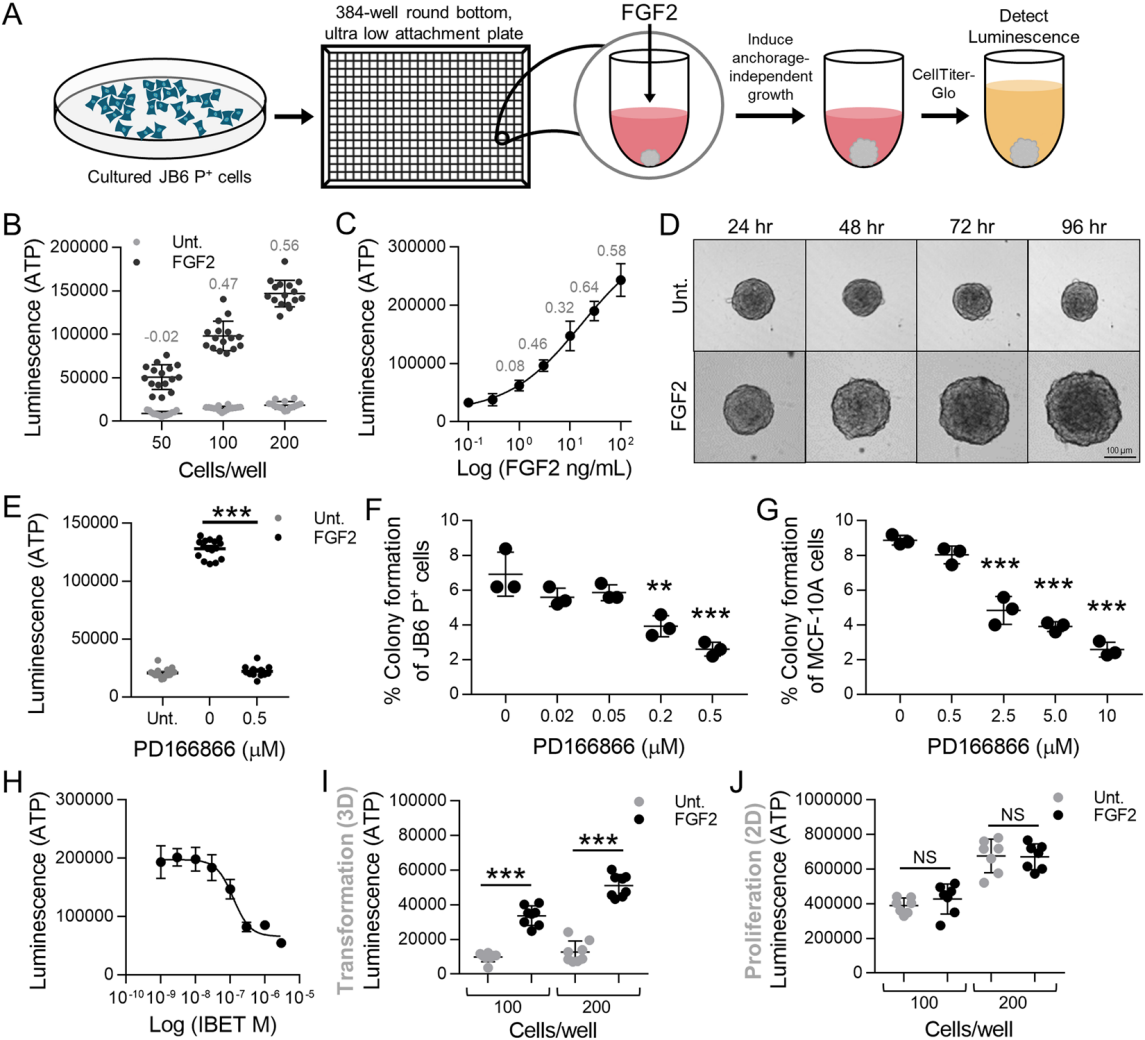
Optimization and development of the transformation HTS. (**A**) Methodology schema of the transformation HTS. JB6 P^+^ cells were plated in 384-well round bottom ultra-low attachment conditions and stimulated with FGF2. JB6 P^+^ cell growth in ultra-low attachment conditions was measured with CellTiter-Glo that gives a luminescent signal stimulated by binding to ATP. ATP levels are proportional to the number of cells, and thus used as a measure of proliferation. (**B**) JB6 P^+^ cells were plated in 384-well round bottom, ultra-low attachment plates and treated with 30 ng/mL of FGF with either 50, 100, or 200 cells/well. 200 cells/well was the optimal cell density giving a Z-factor of 0.56. Fifty (50) and 100 cells/well gave Z-factors of −0.023 and 0.47 respectively. Each treatment group had 16 technical replicates. (**C**) A concentration response of FGF2 was performed with JB6 P^+^ cells at 200/cells per well. The EC_50_ was 15 ng/mL, however, 30 ng/mL gave the optimal Z-factor 0.644. Each treatment had 7 technical replicates. The concentration response stimulation was statistically analyzed using a nonlinear regression, dose-response with PRISM. (**D**) FGF2-stimulated transformation of JB6 P^+^ cells can be visually observed with JB6 P^+^ cells at 200 cells/well with or without FGF2 at 30 ng/mL over the 96-hour incubation. JB6 P^+^ cells congregate at the bottom of the round-bottom wells and untreated, do not grow, but with FGF2, do proliferate. (**E**) PD166866, a FGFR1 inhibitor, at 0.5 μM completely prevented FGF2-stimulated JB6 P^+^ cells growth in ultra-low attachment conditions. Untreated (Unt.) and FGF2 controls had 16 technical replicates and PD166866 had seven technical replicates. (**F**) PD166866 attenuates FGF2 (0.5 ng/mL)-stimulated JB6 P^+^ cell growth in soft agar. The soft agar assay was performed as described in Material/Methods. (**G**) PD166866 attenuates FGF2 (20 ng/mL)-stimulated MCF-10A cell growth in soft agar. The soft agar assay was performed as described in Material/Methods. Data are presented as mean ± S.D., statistical significance was determined using a one-way ANOVA, multiple comparisons (**p < 0.01, ***p < 0.0001). (**H**) IBET concentration-dependently inhibits FGF2 stimulated growth in ultra-low attachment conditions. JB6 P^+^ cells were plated at 200 cells/well with FGF2 at 30 ng/mL. The IC50 of IBET inhibition is 0.12 µM. Each treatment had seven technical replicates. The concentration response inhibition was statistically analyzed using a nonlinear regression, dose-response with PRISM. (**I**) JB6 P^+^ cells were plated in 384-well round-bottom, low attachment plates (100 and 200 cells/well) and incubated at 37 °C for 48 hrs. FGF2 at 30 ng/mL significantly stimulates JB6 P^+^ cell growth in low attachment conditions compared to untreated controls (Unt.). Each treatment had seven technical replicates and data was analyzed by one-way ANOVA. Data are presented as mean ± S.D. (**J**) JB6 P^+^ cells were plated in 384-well flat-bottom, cell culture treated plates (100 and 200 cells/well) and incubated at 37 °C for 48 hrs. FGF2 at 30 ng/mL did not stimulate growth in 2D culture conditions compared to untreated controls (Unt.). Each treatment had seven technical replicates and data was analyzed by one-way ANOVA. Data are presented as mean ± S.D. (**p < 0.01, ***p < 0.0001) NS, not significant.

The transformation HTS was developed using JB6 P^+^ mouse skin epithelial cells. JB6 P^+^ cells cannot proliferate in an anchorage-independent manner, but they have the ability to transform upon treatment with tumor promoters^18^. JB6 P^+^ cells are used below passage 15 to prevent spontaneous transformation. We previously demonstrated that visceral adipose tissue (VAT)-derived FGF2 stimulates JB6 P^+^ cell growth in soft agar (transformation)^5^. FGF2 was used in the HTS assay to stimulate transformation. Using mouse fat tissue filtrate (MFTF), a filtrate that contains factors from the VAT of high-fat diet (HFD)-fed mice, might be more physiologically relevant and constitute a screen with better face validity to identify chemopreventive compounds in obesity-associated transformation. However, using biological matrices in HTS often result in significant variability. Therefore, the assay was developed and optimized with FGF2 as the stimulus to increase rigor and reproducibility. We used CellTiter-Glo (Promega) to quantitate the ATP content in the cells, a surrogate marker for proliferation, as the amount of ATP is generally proportional to the number of cells (Fig. 1A).

The transformation HTS assay parameters, including number of cells plated per well, incubation time, and FGF2 concentration were optimized. To determine the optimal cell number, JB6 P^+^ cells were plated in 384-well round-bottom, ultra-low-attachment plates at 50, 100, and 200 cells/well with FGF2 at 30 ng/mL. 200 cells/well gave an optimal z-factor of 0.56 (Fig. 1B). Using 200 cells/well, a concentration response study was performed. FGF2 at 30 ng/mL gave the optimal z-factor of 0.64 (Fig. 1C). The highest concentration of FGF2 (100 ng/mL) increased the variability, decreasing the Z-factor (Fig. 1C). Figure 1D illustrates FGF2-stimulated growth over a 96-hour period, whereas untreated or vehicle-treated JB6 P^+^ cells congregate at the bottom of the plate and fail to proliferate (Fig. 1D). Overall, the transformation HTS was developed in a 384-well, round bottom, ultra-low attachment plates and optimized to have 200 JB6 P^+^ cells/well, 30 ng/mL of FGF2, 96-hour incubation at 37 °C, resulting in a Z-factor of 0.503 over eight independent assays. DMSO, the solvent for the compound libraries, was used below 0.05% because higher concentrations negatively impacted the Z-factor. Therefore, the compounds were screened with a final DMSO concentration of 0.025%.

### FGFR1 is critical in FGF2-stimulated transformation

The transformation HTS was developed to be a FGFR1 target-based, phenotypic screen. Therefore, this assay can identify hits that act directly on FGF2 and/or FGFR1, as well as ones that target the transformation process, including any part of the FGF2/FGFR1 signaling axis that promotes transformation. To demonstrate that FGFR1 is critical in FGF2-stimulated growth in ultra-low attachment conditions, we used PD166866, an FGFR1-selective inhibitor (SelleckChem), in the screen as a positive control. PD166866 at 0.5 µM completely inhibited FGF2-stimulated transformation in the HTS (Fig. 1E), demonstrating that compounds which inhibit FGF2/FGFR1 signaling will inhibit JB6 P^+^ cell transformation. This result was validated in soft agar with both JB6 P^+^ and MCF-10A cells. PD166866 significantly inhibited JB6 P^+^ colony formation at 0.2 and 0.5 µM and MCF-10A colony formation at 2.5, 5.0 and 10 µM (Fig. 1F,G). These data demonstrate that FGFR1 is critical in FGF2 stimulated epithelial cell transformation.

To demonstrate efficacy and feasibility of this assay, we also tested I-BET-762 (IBET), a bromodomain inhibitor, which we have previously shown to prevent FGF2-stimulated transformation both *in vitro* and *in vivo*^21^. IBET concentration-dependently prevented FGF2-stimulated growth in low attachment conditions (Fig. 1H).

### 2D vs. 3D growth

Cell growth in 2D culture (proliferation) is not mechanistically analogous to cell growth in 3D culture (transformation)^19,20^. To demonstrate this, JB6 P^+^ cells were plated in conventional 384-well flat-bottom, cell culture plates at 100 or 200 cells/well, with or without FGF2 (30 ng/mL) for 48 hours (Fig. 1D). JB6 P^+^ cells were also plated in 384-well round-bottom, ultra-low attachment plates at 100 or 200 cells/well, with or without FGF2 (30 ng/mL) for 48 hours (Fig. 1I). 96 hrs in 2D growth was not optimal because all cells are proliferating and reach confluence before the 96 hours, a limitation that does not apply to ultra-low attachment cell growth because the cells are not restrained to the surface area of the well. FGF2 significantly stimulated proliferation in 3D conditions and not in 2D conditions with either 100 or 200 cells per well (Fig. 1I). FGF2 is a known mitogen that stimulates growth of cancer cells in 2D culture^20^. However, in our assay with non-tumorigenic cells, FGF2 is not a mitogen as proliferation of JB6 P^+^ was not increased by FGF2 (Fig. 1J). This corresponds to findings from Rotem *et al*., which demonstrate that the oncogenic capacity of cells (growth in 3D) is independent of the proliferation rate^16^. Therefore, FGF2 stimulates anchorage-independent growth, a characteristic of malignant cells, which suggests that FGF2 stimulates JB6 P^+^ cell transformation but does not enhance traditional 2D proliferation.

### Screening of compound libraries

Over a thousand compounds from the Prestwick Chemical Library®, the National Cancer Institute (NCI) Natural products library, and the Michigan State University (MSU) Chemistry library of MSU-made analogs of natural products were screened. These libraries were used at 0.5 µM, a relatively low concentration to identify chemopreventive agents with a higher potency and to reduce potential for toxicity. The screen gave an average Z-factor of 0.503. Compounds that attenuated transformation 50% or more (which is 3–4 standard deviations from the mean) were considered primary hits. Compounds were then assessed for general cytotoxicity and eliminated if HEK293 cell viability was decreased by more than 25% at 10 µM (MSU screening core, unpublished). Next, compounds were assessed for promiscuity and eliminated if they demonstrated activity in more than 20% of the bioassays listed in PubChem, determined by number of hits/total assays screened (data acquired in August 2017). Promiscuous compounds are problematic for chemoprevention due to the increased potential for side-effects. These parameters narrowed hits down to 58 compounds for validation and concentration response (Table 1).

**Table 1:** Funnel Strategy −2,532 compounds screened.

Hit Parameter	Identified Hits	Hit Percentage
Transformation HTS −>50% inhibition	178	7.0
Cytotoxicity −<25% decrease in HEK293 cell viability)	105	4.2
Promiscuity - active in <20% assays listed in Pubchem	58	2.3
Commercially available compounds - DTP/CC	33	1.3
Fresh powder confirmation −>40% inhibition	7	0.27

Abbreviations: HEK293, human embryonic kidney; DTP, Developmental Therapeutics Program; CC, Cayman Chemical.

### Hit confirmation and soft agar validation

**Figure 2:**
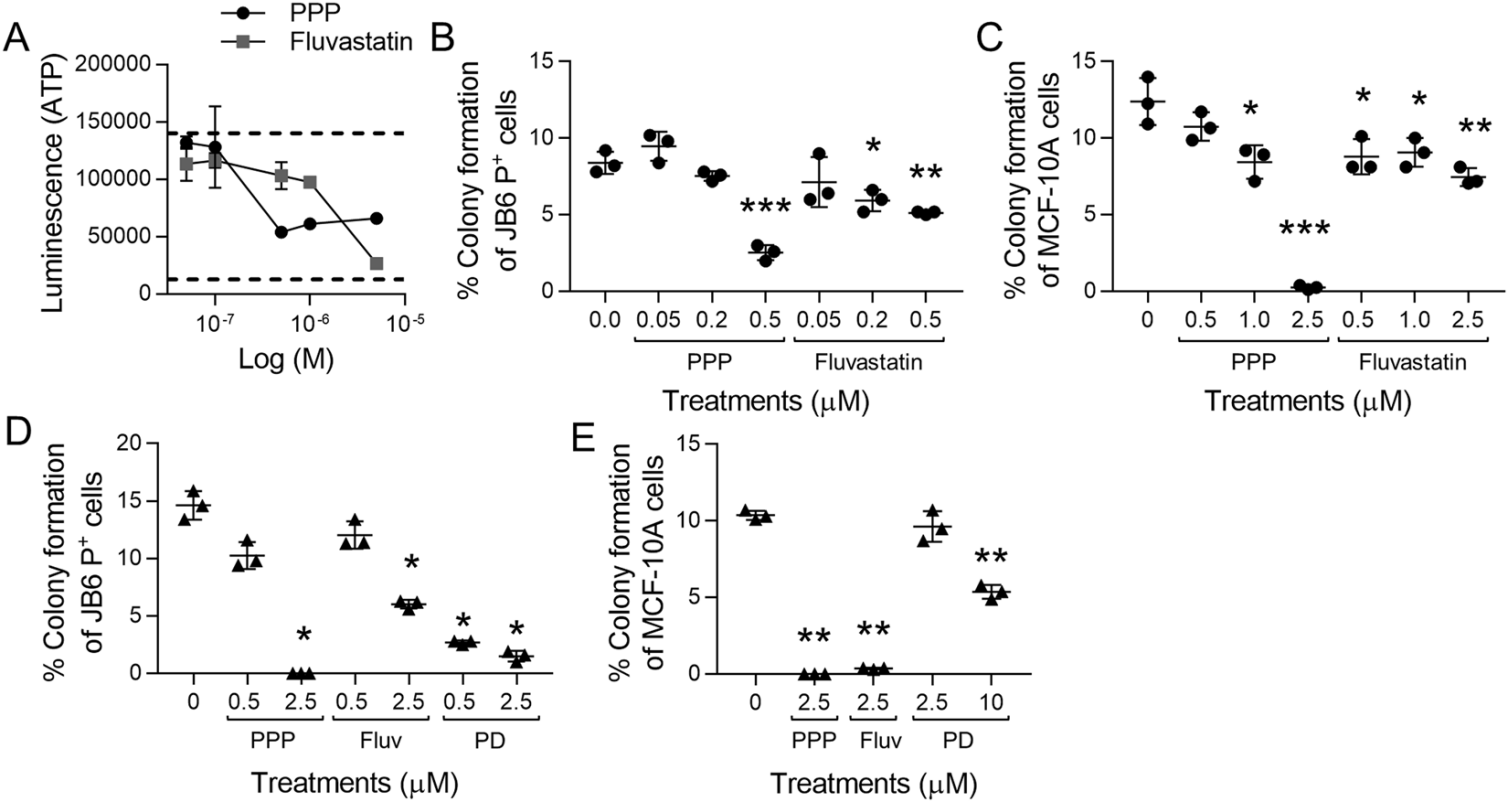
PPP and fluvastatin significantly attenuate FGF2-stimulated transformation of epithelial cells. (**A**) PPP and fluvastatin concentration-dependently inhibit JB6 P^+^ cell growth in low attachment conditions. Dotted lines indicated FGF2 (top) and untreated controls (bottom). Each treatment had three technical replicates. JB6 P^+^ cells were cultured with the optimized parameters. (**B**) PPP and fluvastatin attenuate FGF2 (0.5 ng/mL)-stimulated JB6 P^+^ cell growth in soft agar at 0.5 µM and 0.2 and 0.5 µM respectively. The soft agar was performed as described in Materials/Methods with three technical replicates. (**C**) PPP and fluvastatin attenuates FGF2 (20 ng/mL)-stimulated MCF-10A cell growth in soft agar at 1.0 and 2.5 µM and 0.5, 1.0, and 2.5 µM respectively. The soft agar was performed as described in Materials/Methods with three technical replicates. (**D**) PPP and fluvastatin (Fluv) significantly attenuate MFTF (200 µg/mL)-stimulated JB6 P^+^ cell growth (1000 cells/well) in soft agar at 2.5 µM and PD166866 (PD) was significant at 0.5 and 2.5 µM. The soft agar was performed as described in Materials/Methods with three technical replicates. (**E**) PPP and fluvastatin (Fluv) significantly attenuate MFTF (200 µg/mL)-stimulated MCF-10A cell growth (1000 cells/well) in soft agar at 2.5 µM and PD166866 (PD) was significant at 10 µM. The soft agar was performed as described in Materials/Methods with three technical replicates. The soft agar assays were analyzed by one-way ANOVA with multiple comparisons (*p < 0.05; **p < 0.01; ***p < 0.001).

Fresh powder was obtained from the Developmental Therapeutics Program (DTP) at the NCI or commercially (Cayman Chemical) for 33 compounds to confirm hits and eliminate false positives. Not all 58 prioritized hits were commercially available for order. Confirmation tests with new powders ensure that inhibition of FGF2-stimulated transformation corresponds to the intact compound and not to an impurity or degraded compound in DMSO library stocks which can occur if compounds have undergone several freeze-thaw cycles over time^22^. Of the prioritized hits from the transformation HTS, 2 hits were confirmed: picropodophyllin (PPP) and fluvastatin. PPP is an insulin-like growth factor-1 receptor (IGF1R) inhibitor that is currently undergoing clinical trials as an adjuvant chemotherapeutic. PPP concentration-dependently attenuated FGF2-stimulated growth in low attachment conditions (Fig. 2A). Fluvastatin, a statin drug used for lowering blood cholesterol and triglycerides, concentration-dependently attenuated FGF2-stimulated growth in low attachment conditions (Fig. 2A). Both PPP and fluvastatin have been used clinically with no observed toxicity^23–26^.

PPP and fluvastatin were validated in the soft agar assay using both JB6 P^+^ and MCF-10A cells to demonstrate their efficacy in preventing FGF2-stimulated transformation. MCF-10A cells are non-tumorigenic human mammary epithelial cells. We previously showed that MFTF and FGF2 can stimulate MCF-10A cells to transform^6,21^. Here, PPP and fluvastatin concentration-dependently attenuated FGF2-stimulated colony formation of JB6 P^+^ (Fig. 2B) and MCF-10A cells in soft agar (Fig. 2C), analogous to the inhibition observed in the HTS. JB6 P^+^ cells were stimulated with 0.5 ng/mL of FGF2 and PPP significantly attenuated colony formation at 0.5 µM compared to the FGF2-treated control; fluvastatin also significantly attenuated colony formation at 0.2 and 0.5 µM (Fig. 2B). MCF-10A cells were stimulated with 20 ng/mL of FGF2 and PPP significantly attenuated colony formation at 1.0 and 2.5 µM; fluvastatin significantly attenuated colony formation at 0.5, 1.0, and 2.5 µM (Fig. 2C). Overall, these data demonstrate that compounds discovered through the transformation HTS also inhibit colony formation in the gold-standard soft agar transformation assay.

The objective of these studies is to identify chemopreventive compounds for obesity-related cancers. Therefore, we tested the efficacy of PPP and fluvastatin for preventing MFTF-stimulated transformation. Both PPP and fluvastatin significantly attenuated colony formation at 2.5 µM when JB6 P^+^ cells were stimulated with 200 µg/mL of MFTF (Fig. 2D). The FGFR1 inhibitor PD166866 also significantly attenuated colony formation at 0.5 and 2.5 µM (Fig. 2D). Both PPP and fluvastatin significantly attenuated colony formation at 2.5 µM when MCF-10A cells were stimulated with 200 µg/mL of MFTF (Fig. 2E). The FGFR1 inhibitor PD166866 also significantly attenuated colony formation at 10 µM (Fig. 2E).

### Picropodophyllin and fluvastatin do not inhibit 2D proliferation

**Figure 3:**
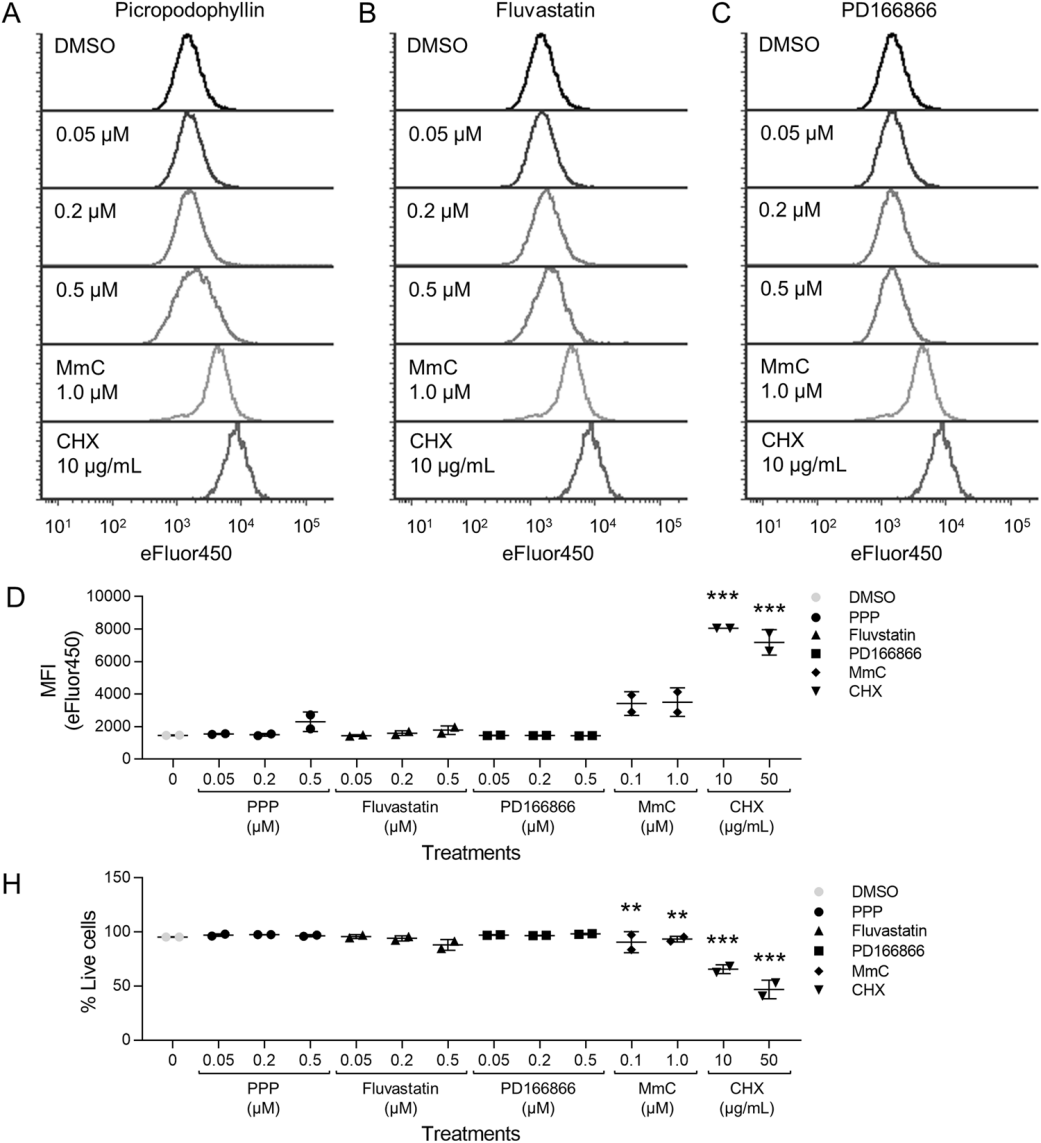
PPP, fluvastatin or PD166866 do not inhibit 2D proliferation of JB6 P^+^ cells. Histograms of JB6 P^+^ cells stained with Cell Proliferation Dye eFluor™ 450 were treated with (**A**) PPP, (**B**) fluvastatin, PD166866 (**C**) or the vehicle, DMSO. Inhibition of proliferation is indicated by higher fluorescent signal that is represented by visually distinct rightward shifted histograms as shown following treatment with mitomycin C (MmC) and cycloheximide (CHX). The same histograms for DMSO, MmC (1.0 µM), and CHX (10 µg/mL) are shown for comparison with PPP, fluvastatin, and PD166866. (**D**) The eFluor™ 450 MFI of JB6 P^+^ cells (singlet, SYTOX Red negative cells) stained with Cell Proliferation eFluor™ 450 and treated with PPP, fluvastatin, or PD166866 was graphically depicted. PPP, fluvastatin, and PD166866 did not significantly influence the eFluor™ 450 MFI compared to vehicle (DMSO) controls, whereas MMC and CHX (positive controls) had significantly higher eFluor™ 450 MFI values. (**E**) PPP, fluvastatin, or PD166866 did not significantly decrease the % of live cells (singlet, SYTOX Red negative cells). MmC and CHX significantly induced cell death at 0.1 and 1.0 µM and 10 and 50 µg/mL respectively. Each treatment group had two replicates and analyzed by one-way ANOVA with multiple comparisons (**p < 0.01; ***p < 0.001).

Many anti-cancer agents inhibit cell proliferation and induce cell death, however, for chemoprevention, compounds need to exhibit little to no toxicity. To determine if PPP and fluvastatin affected cell proliferation and viability. JB6 P^+^ cells were labeled with Cell Proliferation Dye eFluor™ 450 and plated in 2D culture with PPP, fluvastatin, PD166866, or DMSO for 48 hours and analyzed by flow cytometry. The eFluor™ 450 dye binds to cellular proteins containing primary amines. As the cells divide, the dye evenly distributes to both daughter cells. Reduction of the median fluorescence intensity (MFI) by approximately half indicates a successful division. Therefore, cells treated with compounds that inhibit proliferation would have a higher eFluor™ 450 MFI, represented by a rightward shift of the histogram compared to the lower MFI measured in cells that have undergone proliferation. PPP, fluvastatin, and PD166866 had minimal to no effect on JB6 P^+^ cell proliferation in 2D culture, as there was no significant difference observed in eFluor™ 450 MFI between these treatments at any concentration and vehicle (DMSO) treatment. (Fig. 3A–C). Mitomycin C (MmC), a chemotherapeutic agent that inhibits DNA synthesis, and cycloheximide (CHX), a protein synthesis inhibitor, are shown as positive controls. The same histograms for vehicle (DMSO), MmC at 1.0 µM, and CHX at 10 µg/mL are shown for comparison with PPP (Fig. 3A), fluvastatin (Fig. 3B), and PD166866 (Fig. 3C) demonstrating the substantial shift expected when cell proliferation is strongly inhibited. The eFluor™ 450 MFI of the histograms were quantified in Fig. 3D.

All eFluor™ 450 labeled samples were also stained with SYTOX Red dead cell stain at the end of the 48-hour incubation before flow cytometric acquisition of samples to determine whether any of the compounds induced cell death. PPP, fluvastatin, and PD166866 did not significantly induce cell death, as the percent of SYTOX Red negative cells were similar to DMSO vehicle (Fig. 3H). Both MmC and CHX significantly induce cell death compared to DMSO-treated controls (0 µM), as indicated by reduction in the percent of SYTOX Red negative JB6 P^+^ cells. Overall, PPP, fluvastatin, and PD166866 did not influence JB6 P^+^ cell division or cell viability.

### Efficacy of statins and IGF1R inhibitors

**Figure 4:**
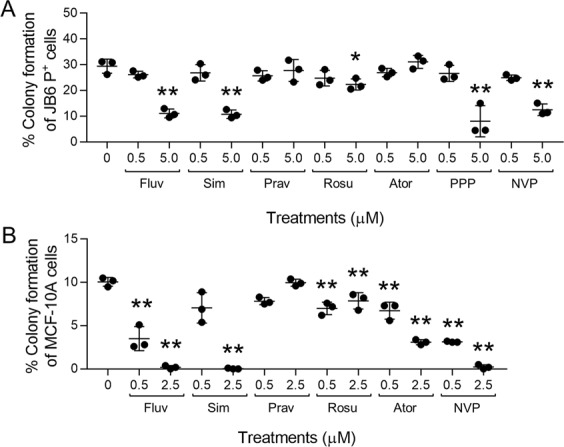
IGF1R inhibitors prevent FGF2-stimulated transformation whereas efficacy of HMG-CoA reductase inhibitors varies. (**A**) Fluvastatin (Fluv), simvastatin (Sim), rosuvastatin (Rosu), PPP, and NVP-ADW742 (NVP) significantly attenuated FGF2 (5 ng/mL)-stimulated transformation of JB6 P^+^ cells (2,000 cells/well) at 5.0 µM. Colonies were counted via automated counting using the Cytation 3 imaging reader from Biotek using Gen5 3.04 software. Seven pictures were taken every 100 microns and superimposed together by the zprojection function. (**B**) Fluvastatin (Fluv), rosuvastatin (Rosu), atorvastatin (Ator), and NVP-ADW742 (NVP) significantly attenuated FGF2 (30 ng/mL)-stimulated transformation of MCF-10A cells (1,000 cells/well) at 0.5 and 2.5 µM. Simvastatin (Sim) was significant at 2.5 µM. Each treatment group had three replicates and analyzed by one-way ANOVA with multiple comparisons (*p < 0.05; **p < 0.01).

To determine if the mechanisms identified by PPP and fluvastatin were compound-specific or applicable to similar classes of drugs, NVP-ADW742 (NVP), an IGF1R inhibitor, and 4 statins (simvastatin, pravastatin, rosuvastatin, atorvastatin) were evaluated for efficacy for preventing FGF2-stimulated transformation. PPP and NVP both inhibited FGF2-stimulated significantly attenuated colony formation. For JB6 P^+^ cells stimulated with 5 ng/mL of FGF2, both PPP and NVP significantly attenuated colony formation at 5.0 µM (Fig. 4A). For MCF-10A cells stimulated with 30 µg/mL of FGF2, NVP significantly attenuated colony formation at 0.5 and 2.5 µM (Fig. 4B). Fluvastatin, simvastatin, and rosuvastatin significantly inhibited FGF2-stimulated JB6 P^+^ colony formation at 5.0 µM (Fig. 4A). Fluvastatin, rosuvastatin, and atorvastatin significantly prevented FGF2-stimulated MCF-10A colony formation at 0.5 and 2.5 µM (Fig. 4B). Simvastatin significantly prevented FGF2-stimulated MCF-10A transformation at 2.5 µM (Fig. 4A).

## Discussion

Primary prevention for high-risk patients is an effective tool for reducing cancer burden. More effective chemotherapeutics and precise molecular targeting have contributed to a substantial decrease in worldwide mortality from 2005 to 2015^27^. In contrast, cancer incidence has steadily increased from 2005 to 2015, rising 33% and affecting more than 14 million people in 2015^27,28^. The cancers investigated in this manuscript, breast cancer and skin cancer, are two of the ten cancers that are still increasing in incidence^29,30^. Interestingly, obesity-associated cancers are preferentially on the rise, and account for 40% of cancers diagnoses^1^. These epidemiological studies illustrate that cancer is still a major health burden and suggests a need to prevent obesity-associated cancer incidence. We previously demonstrated a role for FGF2/FGFR1 in visceral obesity-associated epithelial cell transformation^31^. Therefore, using this mechanism, we aimed to develop an HTS method to screen for compounds that have the potential to prevent or attenuate FGF2/FGFR1-stimulated transformation. Herein we describe the first HTS assay that models the process of transformation in an *in vitro* experimental setting. This transformation HTS assay is a tool to identify potential prevention strategies targeting obesity-associated epithelial cancers and if successful, could greatly reduce cancer burden.

The transformation HTS is a novel FGF2/FGFR1 target-based, phenotypic screen that can evaluate the ability of compounds to inhibit transformation. Target-based drug discovery may lack clinical efficacy because inhibiting a single target doesn’t account for redundancy and/or compensatory crosstalk that may negate target inhibition^32^. Additionally, using phenotypic approaches with no mechanism of action can identify non-selective agents that influence a plethora of processes by acting on other cell types, receptors, or pathways. A screen that utilizes both target-based and phenotypic qualities creates a mechanism-informed, phenotypic screen that overcomes the individual limitations of each screen type and thus can identify compounds that are more likely to be efficacious *in vivo*^32^. The transformation HTS can identify hits that act directly on FGF2 and/or FGFR1 or can target critical factors in the FGF2/FGFR1 signaling axis required to stimulate transformation. This HTS can identify potential chemopreventive agents and serve as a tool to further elucidate the mechanisms of transformation. PD166866, a selective FGFR1 inhibitor, inhibited FGF2-stimulated transformation, in both ultra-low attachment conditions and the soft agar assay, which confirms FGFR1 as the critical receptor in FGF2-stimulated transformation (Fig. 1E–G). Furthermore, the transformation HTS identified PPP and fluvastatin as effective chemopreventive agents (Fig. 2). However, based on our knowledge based on published literature, these two compounds do not directly interact with FGF2 or FGFR1. Therefore, compounds like PPP and fluvastatin can be used to gain mechanistic understanding on how FGF2/FGFR1 stimulates transformation. Screening more compound libraries should reveal more efficacious compounds that would further aid in elucidating the mechanisms of obesity-associated transformation.

The transformation HTS method is the first to stimulate a non-tumorigenic cell to transform *in vitro* while overcoming limitations of the soft agar assay and 2D cell proliferation/apoptosis assays for chemoprevention drug discovery. The soft agar assay in its traditional 6–24 well plate format is laborious, inefficient, and costly, and is not usable for high-throughput screening^5,33,34^. However, studies have scaled up the soft agar assay to a 96– or 384-well format to screen for chemotherapeutic compounds^35–37^. For example, Horman *et al*. developed an HTS-compatible 3D colony formation assay in a 384-well plate by incubating 150 HCT116 human colorectal carcinoma cells with compounds for 5 days, quantifying colonies with a laser-scanning fluorescence cytometer^36^. Methods such as this successfully identify chemotherapeutics but are not suitable for identifying chemopreventive agents for two reasons. First, these soft agar assays use cancer cell lines which are functionally different from non-transformed cells regarding activated/inhibited signaling pathways, changing the druggable pathways in each cell type. Because the methodology for true prevention assays is underdeveloped, inhibiting colony formation of transformed cells is used to investigate both chemotherapeutic^38^ and chemopreventive^39^ compounds, highlighting a need for more effective models for chemoprevention that target the transformation process. Second, using non-transformed cell lines in a high-throughput soft agar assay results in only a small fraction of these cells forming colonies over the course of 10–14 days making visualizing colony formation and inhibition a challenge. Furthermore, achieving a Z-factor of 0.5 or above would be highly improbable. While traditional 2D prevention assays are advantageous because they are easily scaled up to 384–1536-well formats, mechanisms of 2D growth are different from mechanisms of 3D growth^20^. For example, Fig. 1I,J demonstrates that FGF2 stimulates 3D growth of JB6 P^+^ cells, but not 2D growth. Moreover, these traditional 2D prevention assays use cancer cells. Using cancer cells to gain mechanistic insight may not be fully representative of the mechanism(s) to prevent carcinogenesis^38,39^.

One limitation to the transformation HTS, as with many anti-cancer screens, is that cytotoxic compounds will show up as hits. For example, Roridin A and CHX, cytotoxic compounds that inhibit protein synthesis, inhibited 3D growth by more than 60% in the initial screen and were selected as primary hits. Since our libraries consisted of known and/or FDA approved compounds, those compounds could be eliminated based on published/known toxicities in non-transformed cells. CHX was used in the eFluor™ 450 proliferation experiment as a control that inhibits 2D proliferation (Fig. 3). For unknown or novel compounds, following cytotoxicity studies should be performed to ensure that compounds are preventing the process of transformation and are not simply inducing cell death or inhibiting vital cell functions like protein synthesis.

Our transformation HTS resulted in the identification of picropodophyllin (PPP) as an inhibitor of FGF2-stimulated growth in ultra-low attachment conditions. PPP is a cyclolignan alkaloid from the mayapple plant family. PPP has been suggested to have anti-neoplastic activity by inhibiting IGF1R^40^, a receptor tyrosine kinase that is a key regulator of energy metabolism ant tumor growth. PPP inhibits the IGF1R by inducing the activation loop-specific inhibition of tyrosine phosphorylation. Although it has been suggested that PPP is specific for IGF1R at nanomolar concentrations, it is unknown if PPP at micromolar concentrations will inhibit FGF1R, a mechanism that will be explored in future studies. A role of IGF1R itself in transformation is supported by recent investigations into metformin as a chemopreventive agent. Metformin inhibits insulin like growth factor 1 (IGF1)/IGF1R signaling^41^. Furthermore, an additional IGF1R inhibitor, NVP-ADW742 attenuated FGF2-stimulated transformation of JB6 P^+^ cells suggesting a potential role for IGF1R in FGF2/FGFR1-driven transformation. Interestingly, there are elevated circulating and tissue levels of both insulin growth factor and insulin in obesity, suggesting that a combination of elevated growth factors may increase cancer risk^25,41^.

PPP has demonstrated both safety and efficacy in clinical studies and mouse models of tumorigenesis. In a phase I/II trial of four patients with squamous cell lung carcinoma, PPP treatments induced necrosis in the tumor and disease progression was halted for seven months^23^. None of the patients in this study showed dose-limiting toxicity^23^. These studies showed that PPP is a potential chemotherapeutic and has good tolerability^23^. *In vivo* mouse models demonstrated that PPP decreased tumorigenesis with no associated toxicity. In a mouse model of Benzo(a)pyrene (BaP)-induced lung tumorigenesis, PPP decreased tumor volume, increased apoptosis (caspase-3) and decreased proliferation (Ki-67) in the tumor^24^. Additionally, these A/J mice were treated with PPP once a day, five times a week for 20 weeks and there were no changes in body weight and no overt side effects^24^. In another study using a xenograft model of multiple myeloma (MM), PPP was subcutaneously administered to mice with established MM tumors. PPP significantly decreased tumor burden and inhibited tumor- associated angiogenesis and osteolysis. PPP also significantly prolonged the life of the mice from 100 days to 150 days^25^. It is important to note that current published studies examine effects of PPP on established tumors, whereas this manuscript investigates PPP as a chemopreventive agent, we test its ability to prevent the process of transformation, revealing a new clinical target for prevention that has not been previously explored. Collectively, these studies show that PPP has oral clinical efficacy in humans and overall is well tolerated, suggesting that PPP has the potential to have utility for cancer prevention.

Our transformation HTS also identified fluvastatin as an inhibitor of FGF2-stimulated growth in ultra-low attachment conditions in the primary screen. Fluvastatin is one of several 3-hydroxy-3-methylglutaryl coenzyme A (HMG-CoA) reductase inhibitors, cholesterol lowering agents that treat dyslipidaemia and prevent cardiovascular disease^42^. Statins work by competitive inhibition of HMG-CoA reductase, the rate-limiting step in cholesterol biosynthesis, causing reductions in cholesterol and low-density lipoproteins (LDL) and an increase in high-density lipoproteins (HDL), that carry cholesterol from other parts of the body to the liver for removal^42,43^. Fluvastatin is a good candidate for chemoprevention because it has a favorable safety profile and has been shown to have anti-cancer activity^43^. Fluvastatin inhibits breast cancer cell proliferation and with a greater potency in estrogen receptor (ER) negative breast cancer cells^44,45^. Interestingly, fluvastatin inhibited FGF2-stimulated transformation of MCF-10A cells, which are ER negative. Recently, FGFR1 activation was identified as the primary mechanism by which obesity drives estrogen receptor positive mammary tumor progression following endocrine deprivation^46^. These studies suggest that fluvastatin may be efficacious for inhibiting obesity-promoted mammary tumor progression and a potential compound for secondary prevention in obese patients.

Epidemiological studies that evaluate statins and cancer risk have been inconclusive. A 2006 meta-analysis by Browning *et al*. reviewed the association between statins and cancer risk investigating 42 studies and concluded statins use is not associated with short-term cancer risk. However, these studies had relatively short follow-ups that were too brief to capture a true association between statin use and cancer incidence or mortality^47^. In more recent analyses, Yang *et al*.^48^ analyzed four articles and came to the tentative conclusion that fluvastatin may reduce breast cancer risk but further high-quality research is needed to confirm this. Likewise, Liu *et al*.^49^ investigated seven studies and suggested that lipophilic statins (like fluvastatin, simvastatin, and atorvastatin) were more protective than hydrophilic stains (like pravastatin and rosuvastatin) but due to high heterogeneity between the studies made it “difficult to assign a truly advantageous benefit for this population^49^.” Ultimately, long-term data is lacking to support the role of statins in primary chemoprevention^50,51^. We investigated four additional statins including simvastatin, rosuvastatin, pravastatin, and atorvastatin to determine if the effects on colony formation were specific to fluvastatin. Not all the statins attenuated colony formation, nor did efficacy correlate simple with statin lipophilicity. Additionally, similar effects were observed in both cell lines, except for atorvastatin which had no effect on colony formation of JB6 P^+^ cells but attenuated colony formation of MCF-10A cells (Fig. 4). Follow-up studies focused on mechanisms underlying the efficacy of statins are warranted.

Overall, we optimized a novel HTS of FGF2-stimulated transformation utilizing growth in ultra-low attachment conditions. This is the first screen to stimulate non-tumorigenic cells to transform *in vitro*. Additionally, this assay has the potential to be optimized with other tumor promoters such as hepatocyte growth factor, epidermal growth factor, and phorbol esters, as well as with complete carcinogens such as BaP. The transformation HTS identified PPP and fluvastatin as potential chemopreventive agents. After these compounds were confirmed to concentration-dependently inhibit FGF2 stimulated transformation, they were validated in soft agar with two non-tumorigenic cell lines, JB6 P^+^ and MCF-10A cells. Future studies will test these compounds *in vivo* and evaluate their mechanism of action. Overall, the transformation HTS is a fast, robust and uniquely adept 3D screen to identify potential chemopreventive compounds.

## Materials/Methods

### Cell culture

JB6 P^+^ cells (mouse skin epidermal cells) were obtained from ATCC (Manassas, VA, USA). Cells were cultured in minimum essential medium (MEM) supplemented with 5% fetal bovine serum (FBS) and 1% penicillin/streptomycin (p/s) (JB6 P^+^ growth media). JB6 P^+^ cells were trypsinized with 0.05% trypsin and quenched with MEM with 5% FBS and 1% p/s. JB6 P^+^ cells are used below passage 15 prevent spontaneous transformation.

MCF-10A cells (human mammary epithelial cells) were obtained from ATCC (Manassas, VA, USA). Cells were cultured in DMEM/Ham’s F:12 media supplemented with 5% horse serum (HS), 1% p/s, 100 ng/mL cholera toxin, 20 ng/mL epidermal growth factor (EGF), 10 µg/mL insulin, 0.5 mg/mL hydrocortisone, 7.5% sodium bicarbonate, 15 mM HEPES, and 2 mM L-Glutamine (MCF-10A growth media). MCF-10A cells were trypsinized with 0.05% trypsin and quenched in DMEM/ Ham’s F12 media with 20% HS and antibiotics (resuspension media). MCF-10A cells are used below passage 20 prevent spontaneous transformation.

### Reagents

Mouse FGF2 is a recombinant protein purchased from Prospec (CYT-386). Fresh powder for confirmation was ordered from the NCI’s DTP or from Caymen Chemical. Picropodophyllin and fluvastatin were obtained from the DTP (Fig. 2). Fluvastatin (10010337), simvastatin (MK-733), rosuvastatin (ZD 4522), pravastatin (10010342), atorvastatin (10493), cycloheximide (14126), and mitomycin C (11435) were ordered from Caymen Chemical. PD166866 (S8493) and NVP-ADW742 (S1088) was purchased from SelleckChem. eBioscience™ Cell Proliferation Dye eFluor™ 450 (65-0842-85) and SYTOX™ Red Dead Cell Stain, for 633 or 635 nm (S34859) were purchased from ThermoFisher. I-BET-762 was purchased from JSTAR Research Inc.

### Generating fat tissue filtrate

Mouse fat tissue filtrate (MFTF) was made as previously described^31^. Briefly, the parametrial fat pad was removed from 13 week old mice that had been on a HFD for 4 weeks. This fat was placed in a transwell insert above serum free media to collect factors from the fat (MFTF). This fat was used in the soft agar assay at 200 µg/mL.

### Soft agar assay

JB6 P^+^ cells were plated at 500 cells/well (or otherwise indicated) in a 24-well plate in 200 µL of MEM media with 10% FBS in 0.33% agar overlaid onto 350 µL of MEM media with 10% FBS in 0.5% agar. FGF2 (Prospec, CYT-386) was incubated with the cells at 0.5 ng/mL and compared to untreated controls. Soft agar plates were left at room temperature for 30 minutes then incubated at 37 °C.

MCF-10A cells were seeded at 750 cells/well (or otherwise indicated) in a 24-well plate in 200 µL of DMEM/Ham’s F12, 5% HS, and 0.33% agar overlaid onto 350 µL of DMEM/Ham’s F12, 5% HS, and 0.5% agar. FGF2 (prospec, CYT-386) was incubated with the cells at 20 ng/mL and compared to untreated controls. Soft agar plates were left at room temperature for 30 minutes before 200 µL of MCF-10A growth media was gently added to each well and then stored at 37 °C. Every 3–4 days, the growth media was removed from each well and replenished with 200 µL of MCF-10A growth media.

After two weeks, JB6 P^+^ and MCF-10A soft agar plates were fixed in 70% ethanol (EtOH) and stained with 150 µL of 0.01% crystal violet. Colonies were visually counted and used to calculate the percent of colony formation from the number of cells plated ([Colonies counted × 100]/number of cells plated).

### Transformation HTS

JB6 P^+^ cells were plated in 384-well round-bottom low attachment plates at 200 cells/well in 40 µL of JB6 P^+^ growth media. FGF2 (30 ng/mL) was used to stimulate JB6 P^+^ cell growth in ultra-low attachment conditions. Cells were manually added to each plate with a multichannel, repeater pipette. Compounds were plated using a dual arm Biomek FX liquid handling robot with 384-well pintool liquid handling system. Plates were incubated at 37 °C for 96 hours. Then 40 µL of CellTiter Glo (Promega) was added to each well. Plates were shaken at 300 rpm for 5 mins on a plate shaker and then a Biotek synergy Neo HTS Multi-Mode Microplate Reader detected the luminescence signal of each well. Untreated cells were used as the negative control. For all compound screening, FGF2 and untreated controls were treated with the vehicle, DMSO.

A Z-factor was used to evaluate the quality of a HTS. A Z-factor is a screening window coefficient that qualitatively assesses the ability of a screen to identify active compounds or hits while screening large compounds libraries. The Z-factor considers the means and standard deviation of the positive and negative controls (Z factor = 1 − (3(𝜎_p_ + 𝜎_n_)) / | μ_p_ – μ_n_ |). A high quality HTS assay has a Z-factor that ranges between 0.5 and 1.0. A Z-factor was calculated during method development to optimize the parameters of the screen to achieve a Z-factor of 0.5 or above during the screening assay.

### Compound libraries

The Prestwick Chemical Library®, the National Cancer Institute (NCI) Natural products library, and a Michigan State University (MSU) library of MSU-made compounds were used for screening in the MSU Assay Development and Drug Repurposing Core (ADDRC). The Prestwick Chemical Library® is a unique collection of 1280 diverse, small molecules consisting of mostly FDA approved drugs, with known bioavailability and safety. The NCI Natural products library consists of 419 compounds selected from the Developmental Therapeutics Program (DTP) open repository and has a variety of scaffold structures with multiple functional groups. The MSU compounds were synthesized by Dr. Jetze Tepe (MSU) and designed to mimic the diverse structural features found in natural products.

### Cell viability and proliferation assessment by flow cytometry

JB6 P^+^ cells were stained with Cell Proliferation Dye eFluor™ 450, resuspended in JB6 P^+^ growth media and were plated at 50,000 cells/well in 6-well plates. After 24 hours, cells were treated with PPP, fluvastatin, PD166866, or DMSO (vehicle control). After an additional 48 hours, cells were prepped for flow cytometric analysis. Cells were trypsinized, washed, resuspended in PBS, and filtered. Prepared JB6 P^+^ cells were stained with SYTOX ™ Red Dead Cell Stain at a concentration of 5 nM, which was added approximately 15 minutes before analyzing samples on a BD FACS Aria IIu located in the MSU South Campus Flow Cytometry Core. Flow cytometry data was analyzed using FCS Express (DeNovo Software). Viability was assessed as a percentage of singlet, SYTOX Red negative cells. Proliferation was assessed by measuring the eFluor™ 450 median fluorescence intensity (MFI) of live JB6 P^+^ cells (singlet, SYTOX Red negative cells).

### Statistical analysis

Data are presented as mean ± SD. For soft agar experiments, three technical replicates were used and analyzed by one-way ANOVA, multiple comparisons. For HTS method development, Z-factor was used to define optimal parameters giving a quality HTS able to define primary hits. For all statistical tests, the 0.05, 0.01, and 0.001 level of confidence was accepted for statistical significance.

## Data Availability

The datasets generated during and/or analyzed during the current study are available from the corresponding author on reasonable request.
